# Team science training needs and preferences for clinical research professionals

**DOI:** 10.1017/cts.2026.10803

**Published:** 2026-07-16

**Authors:** Carolynn Thomas Jones, Jessica M. Fritter, Bernadette Capili, Nicole Exe, Shirley L.T. Helm, Jacqueline Knapke, Elizabeth Kopras, Jill McCabe, Jack Kues, Meredith B. Fitz-Gerald, Angela Mendell

**Affiliations:** 1 School of Nursing, University of Alabama at Birmingham Health System, Birmingham, AL, USA; 2 Center for Clinical and Translational Science (CCTS), The University of Alabama at Birmingham, Birmingham, AL, USA; 3 College of Nursing, The Ohio State University, Columbus, OH, USA; 4 Clinical Translational Science Institute, The Ohio State University, Columbus, OH, USA; 5 Rockefeller University, New York, NY, USA; 6 University of Michigan, Ann Arbor, MI, USA; 7 C. Kenneth and Dianne Wright Center for Clinical and Translational Research, Virginia Commonwealth University, Richmond, VA, USA; 8 University of Cincinnati, Cincinnati, OH, USA

**Keywords:** Clinical research professionals, team science competencies, training preferences, focus groups, needs assessment

## Abstract

**Background:**

Clinical research professionals (CRPs) are essential members of clinical translational science teams. Team science (TS) training exists for clinical research investigators and trainees; however, such training has been lacking for CRP staff. We sought to better understand the TS training and learning preferences for CRPs using a survey and focus group mixed methods study to inform future training.

**Methods:**

The survey was distributed to CRP institutional networks and to the members of the Association of Clinical Translational Science CRP Taskforce Special Interest Group. Post-survey, focus groups were also conducted with volunteers from the survey to further elucidate CRP TS training preferences, including facilitators and barriers to training.

**Results:**

A total of 193 CRPs completed the survey over 7 weeks. Most respondents were clinical research coordinators or clinical research nurses. CRPs responded that TS competencies were important and frequently used in their roles; however, TS training was lacking. Respondents preferred a variety of training modalities such as interactive case studies, webinars, and micro-learning. Qualitative focus group results validated the survey results and provided additional insights into facilitators and barriers to CRP professional development, particularly time constraints for training.

**Discussion/conclusion:**

This is the first learning needs assessment focusing on CRP TS learning needs and training preferences. Together with recently published CRP TS competencies, this study sets the stage for focused training development to enhance CRP roles and interdisciplinary TS.

## Introduction

Clinical research professional (CRPs) are essential members of clinical research teams and vital to the institutional clinical research infrastructure overall[1–3]. CRPs begin as members of their immediate teams; however, their roles quickly evolve and expand, requiring them to engage and coordinate with personnel across the research enterprise who are crucial for conducting research (e.g., Institutional Review Boards (IRB), laboratories, special procedures and testing departments, nursing units, biostatisticians, data managers, clinicians, early career, and established investigators). Investigators and trainees experience more intentional team science (TS) training, required by CTSA hub workforce development aims and similar to other scientific disciplines [4]. In contrast, CRP understanding and perceptions of team dynamics, roles, and responsibilities are typically learned informally, without intentional TS training or education, and through trial and error. This lack of TS understanding and implementation is often explained by the nature of how CRPs enter the field (unintentional versus deliberate), their initial roles (task-oriented vs. interdisciplinary team membership), and by who and how CRP performance oversight is provided [3]. CRP institutional onboarding is typically based upon institutional policies and processes, including Good Clinical Practice (GCP) and Human Subject Protections (HSP) training (e.g., CITI training) [5], and standard operating procedures (SOPs) with minimal focus on TS principles and professional growth. Nevertheless, the importance of TS competence for CRPs is emphasized in Domain 7 (Leadership and Professionalism) and Domain 8 (Communication and Teamwork) of the Joint Task Force (JTF) Clinical Trial Competencies, which provides a framework that defines knowledge, skills, and abilities necessary for conducting safe, ethical, and high-quality clinical research [6]. Domain 8 of the JTF competency specifically stresses “….the importance of TS and methods are necessary to work effectively with cross-function, multidisciplinary and interprofessional research teams.” [6]

Recognizing this gap in CRP-specific TS learning needs, a collaborative working group developed and published CRP TS competencies based on the Lotrecchiano, et.al. definition of thirteen individual and team competencies (Table 1) [7,8]. The previously published CRP TS competencies were intentionally leveled as fundamental, skilled, and advanced to illustrate progressive acquisition of these TS competencies as CRPs progress through their careers [8]. Following the publication, the working group sought the identification of CRP TS learning needs and training preferences with the goal of setting the stage for developing CRP TS specific training, education, and resources. Table 1. TS competencies used in our survey [7,8].

**Table 1.** CRP team science competencies[7,8] Table 1 long description.

Building trust Meeting management Interdisciplinary collaboration Understanding complexity Team learning, adaptive behaviors Team based communication Shared visioning Passion/perseverance Team roles Self-awareness Interdisciplinary research management Cognitive openness Facilitating awareness and exchange

*Note:* CRP = clinical research professional.

Prior to COVID-19, CRP training and learning were conducted primarily in person, with an inherent perceived value in face-to-face interactions [9]. Online and virtual learning was viewed as supplemental and primarily reserved for specific purposes (e.g., institutional human resources trainings) [5]. However, the COVID-19 pandemic created an abrupt and significant shift within educational, training, and learning landscapes (including CRP training and learning), moving swiftly from in-person formats to remote and virtual modalities [10]. Instructional practices changed dramatically with the incorporation of digital technologies, leading to a burgeoning of instructional delivery and learning management platforms, and diversification of andragogical approaches. Educators and learners alike found advantages within this new landscape with increased flexibility and accessibility, possibilities for curriculum expansion and enrichment, and self-paced learning, among others [10]. Nevertheless, challenges within this learning environment include learner engagement, observed skill and competency demonstrations (often clinically based), and reduced networking and collaboration opportunities.

In the post-COVID-19 pandemic landscape, the shift in learning preferences and delivery methods persists, although blended modalities encompassing online learning for knowledge transfer complemented with in-person skill and competency acquisition and demonstration have gained traction [11]. CRP professional certification exam modalities are in step with this shift as certification exam administration through computer-based testing sites and remote testing is the norm, offering greater accessibility, convenience, and flexibility for exam candidates [12,13].

Effective training programs use an education (learning) needs assessment model to inform learner-centric design and development [14]. A needs assessment is a structured process critical in identifying existing gaps in the current state of knowledge and performance within a specific area (in this case, CRP TS competencies) and the desired state of knowledge and performance [15]. Needs assessments typically consist of five steps: Identification of the Problem, Identification of Data Sources, Data Collection, Data Analysis, and Recommendations [15]. Understanding these factors provides valuable insight into the design and development of sustainable learning and training programs to ensure the curricula and modalities are tailored specifically to address the problem identified rather than relying upon assumptions thereby preventing redundant and ineffective training while maximizing the return on investment for training development and delivery.

## Methods

In 2024, the CRP TS working group published leveled (fundamental, skilled, and advanced) TS competencies for CRPs [8]. In this project, we used a multi-method cross sectional descriptive design including a quantitative online needs assessment survey followed by qualitative focus groups to assess learning gaps, needs, and training preferences of CRPs on TS competencies [16]. The project including informed consent were reviewed and approved as exempt by the University of Cincinnati IRB. Leveraging the needs assessment model, we evaluated CRPs understanding and comprehension of TS, a crucial element in effective and efficient multidisciplinary research teams.

### Survey instrument

We used REDCap, a secure electronic data capture tool hosted at the University of Cincinnati as the survey instrument to gain an understanding of CRP perceptions of their TS competencies and learning needs, using 13 individual and TS competencies [7] applied by the CRPT SIG TS Working Group in defining CRP-specific leveled TS competencies [8]. We endeavored to understand the importance of TS to the CRP’s daily work (Question 1), the frequency of using TS competencies in their work (Question 2), past experience with TS training across the competencies (Question 3); and their perceptions of how beneficial TS training would be in their roles (Question 4). Finally, we sought to understand CRP likelihood of attending different forms of TS training (Question 5). The survey questions and scales are described in Table 2 and were applied to each of the 13 TS competencies listed in Table 1. We also collected general demographic data so that we could describe the respondent population including roles, clinical research work experience, and highest level of education.

**Table 2.** CRP questions and Likert scale per 13 team science competencies[7,8] Table 2 long description.

Question #	Question	Likert scale
1	“How important is it for you in your work to apply these Team Science Competencies?” (Select 1 per competency)*	Very unimportant Unimportant Important Very important Not sure what this Is or Not applicable
2	“How often do you use these team science competencies in your CRP role?” (Select 1 per competency)*	1–2 times per year 1 time per month A couple of times per month Daily, or Not applicable
3	“Have you participated in team science training in these competencies?” (Select 1 per competency)*	Yes No Not sure
4	“How beneficial would CRP Team Science Competency Training be for you?” (Select 1 per competency)*	Not at all beneficial Minimally beneficial, Beneficial Very beneficial, or Not applicable
5	“What is the likelihood of attending these forms of team science training?” *(Select 1 per training modality)* Practical Learning (Interactive Case Studies) Small Group Learning/ Peer-Mentoring ELearning (self-paced online modules) Multi-Session Workshop Series Micro-Learning (5 min videos) Single Workshop In-person, instructor-led training	Very unlikely Unlikely Likely Very likely

*Note:* CRP = clinical research professional; ELearning = electronic or online learning; see Table 1.

#### Survey recruitment and eligibility criteria

Purposive sampling was conducted to obtain the target population of CRPs who were recruited via email from the study team’s institutional professional research networks and the survey invitation and link were also sent to the ACTS CRP Taskforce SIG network members.

### Post-survey focus groups

#### Methods

After completing the survey, participants were invited to volunteer for focus groups to discuss the survey topics. Focus groups were intentionally segmented by years of experience (0 to 7 years and >7 years) to explore potential differences in perspective across career stages. Volunteers provided their names and email addresses. After survey analyses, those who volunteered were sent an email to re-affirm their willingness to participate and to schedule focus group participation.

We planned focus groups of around 7 participants per session, depending on schedules with a goal of 2–5 participants per session. Although smaller than traditional focus groups, this size anticipated busy CRP scheduling constraints and enabled deeper, more detailed discussion. We also allowed for individual interviews due to anticipated last-minute schedule conflicts. A standardized facilitator guide was developed by the focus group (FG) study team and was informed by initial survey findings.

Focus groups were facilitated by members of the FG study team with experience in qualitative methods. Transcripts were de-identified before analysis. Independent coding was conducted by two team members who were not involved in facilitating the focus groups to reduce potential bias.

A focus group guide was developed to ensure that all focus groups were conducted consistently. We planned each session to include 2–5 CRPs and last approximately 90 minutes using Zoom (Zoom Video Communications, 2024). Zoom has been used successfully in qualitative focus group sessions [17]. The semi-structured interview guide covered team dynamics and preferred learning strategies informed by best practices for focus groups [18,19]. Therefore, questions were sequenced from general to specific, emphasizing the core concepts of the study and were structured to help participants ease into the topic and reflect on their experiences and opinions. Also, the focus groups and interviews explored examples of study team dynamics in clinical research. The development of these questions was guided by CRP TS competencies [8] See Table 3 for the list of focus group questions from the FG Facilitators Guide.

**Table 3.** Focus group questions Table 3 long description.

**A.** **Questions Related to Survey Results** *What does team science mean to you? (e.g., Who makes up the research team?)* *The survey showed that respondents needed more information on the following terms: Interdisciplinary research management/collaboration. What does interdisciplinary management and collaboration mean to you?* *Shared visioning was a term that caused some confusion in the group – What is your understanding about “shared visioning”*
**B.** **Questions Related to You and Your Team** *Can you describe a recent situation where your team worked well together? What factors do you think contributed to this success?* *How does your team handle disagreements or conflicts? Can you share an instance where this was demonstrated?* *Can you recall when your team did not work well together? What were the main challenges or issues encountered?* *How did these challenges affect your team’s performance and outcome of the project?* *How do you balance your workload with the need for ongoing training and professional development?* *The survey showed a shift toward online training, live webinars, and asynchronous on-demand approaches. What are your thoughts about those training modalities?*

*Notes:* e.g., in other words.

A facilitator moderated the sessions, and a team member served as a scribe to take notes to capture verbal and nonverbal cues. At the beginning of each session, an eight-minute prerecorded video explaining the TS project was shown. The scribe maintained a “speaker log” and recorded the initial words of each participant’s response. Only first names were used to protect the privacy of participants. The study team addressed participants by name, especially when seeking clarification or elaboration. Using first names can foster group identity and cohesiveness [20].

During discussions, the scribe also documented notable quotes and nonverbal behaviors – such as body language or silent agreement – that might not appear in the transcription [18] Each session began with an introduction to the discussion agenda and an explanation of the ground rules, such as allowing one speaker at a time and maintaining participant anonymity [20]. Participants were also asked to share their current roles and years of experience. Focus groups and interviews were segmented by years of experience, 0–7 years, and greater than 7 years. Due to unforeseen scheduling conflicts, two participants opted for an individual interview, since they missed the focus group session due to unforeseen scheduling conflicts. These two individual interviews allowed further exploration of experiences and preferences. The one-on-one sessions were conducted virtually and lasted between 45 and 60 minutes. The same semi-structured interview guide used as in the focus groups to ensure consistency while allowing flexibility to probe unique insights. Participants consented to have all focus group and interview discussions recorded by Zoom and transcribed verbatim for analysis.

#### Post-focus group and interview activities

After participants left the ZOOM session, the study team conducted a quick 2–3 minute debrief. Following the debriefing, the audio recording of the ZOOM session was securely downloaded to a secure cloud-based server, and the audio quality was reviewed. Once all focus groups and interviews were completed, the recordings were uploaded to Quirkos [21], a cloud-based AI transcription service. The research team verified the transcripts for accuracy. Personal identifiers such as names and institutional affiliations were removed from the transcribed data to ensure confidentiality.

#### Qualitative analysis methods

Focus group and interview data were analyzed using a qualitative content analysis approach, combining both deductive and inductive coding [22]. A preliminary coding framework was developed based on established TS competencies, while allowing for emergent themes from the data.

AI-supported transcription and preliminary coding tools were used to assist in data organization; however, all coding and thematic interpretation were conducted and validated by members of the research team. Two independent coders reviewed transcripts and developed coding structures. Codes were compared, discussed, and refined in structured team meetings, with differences treated as opportunities for deeper analytic engagement rather than for simple resolution, consistent with established guidance that emphasizes reflexivity, dialog, and transparency in team-based qualitative analysis [23]. Thematic saturation was reached as no new themes emerged in later focus groups or interviews.

As the analysis progressed, codes were grouped into broader categories and themes, with attention to both shared patterns and variation across participant roles. Themes were reviewed and refined through ongoing team discussion to ensure they remained grounded in the data. AI-assisted tools Delve (delvetool.com) were used in a limited capacity during the early stages of analysis to support data organization and preliminary pattern identification, reflecting emerging approaches that position AI as a tool for augmenting, rather than replacing, human analytic processes [24]. These outputs were treated as provisional and critically reviewed by the research team, recognizing that AI-generated outputs may lack contextual sensitivity and can reflect underlying biases in training data [25].

The research team led all coding decisions, thematic development, and interpretive judgments. When AI-generated groups did not align with the data or analytic intent, they were revised or discarded. In this way, AI served as a pragmatic aid to early data engagement. At the same time, analytic ownership and interpretive rigor remained firmly grounded in human judgment, consistent with ongoing methodological discussions emphasizing the centrality of human interpretation in qualitative inquiry [23,25].

## Results

### Needs assessment survey

#### Demographics

A total of 193 individual CRPs responded to the survey, and most were clinical research nurses (CRNs), clinical research coordinators (48%), or project/program managers (29%). The survey was available from February 23, 2024 and was closed on April 15, 2024.

Although 35% of respondents had over 10 years of experience, the experiences levels were closely distributed across levels: 0 to 2 years (22%); 3 to 5 years (23%); 6 to 10 years (20%); and >10 years (35%). The following data reveal CRP respondents’ perception of TS learning and training needs. Notably, some respondents failed to answer all these questions, so the tables reveal the numbers of total responses for each question.

#### Learning needs

**Figure 1.**
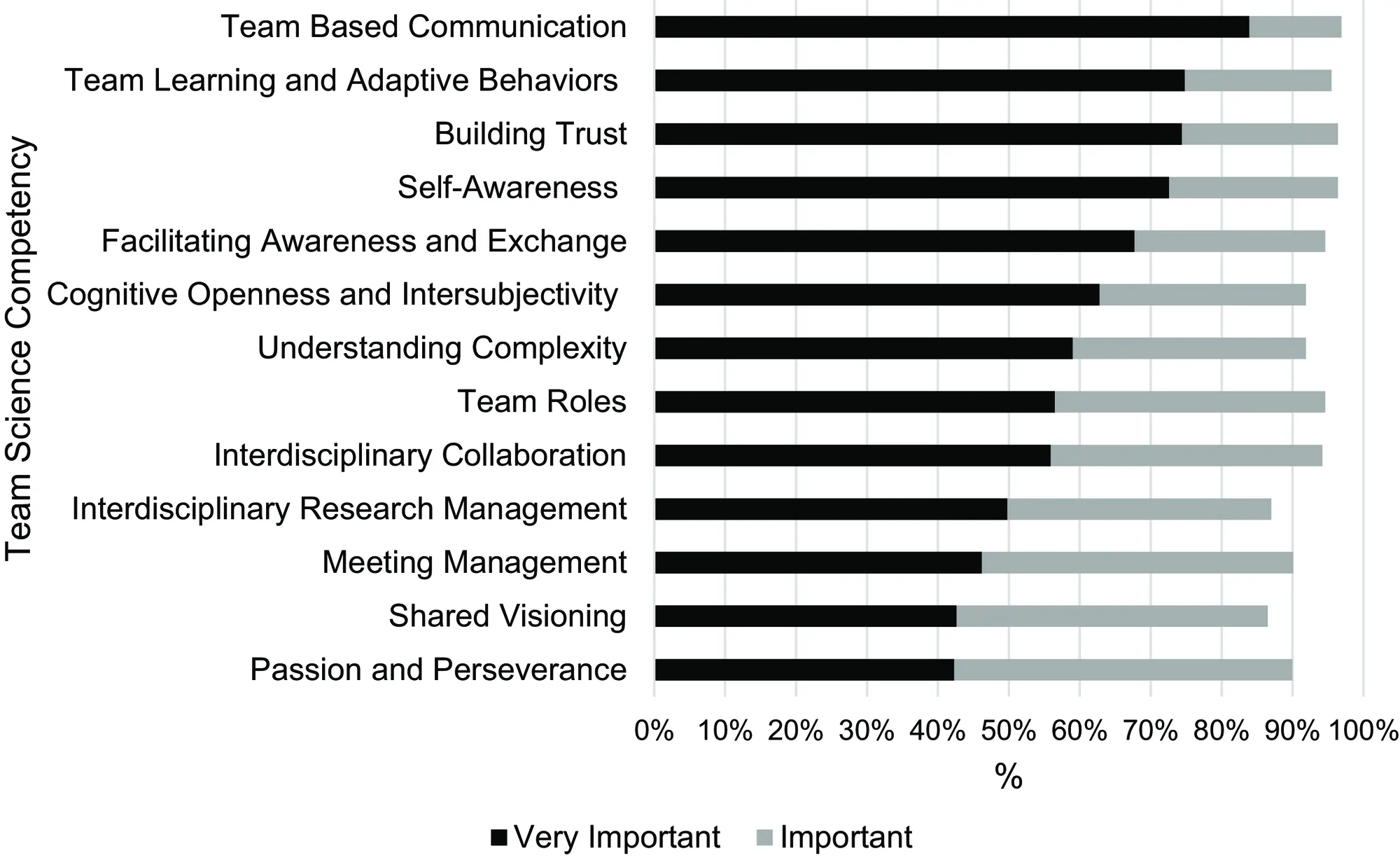
Figure 1 long description. Percentage of CRP respondents’ scores of TS importance (Important, Very important) (*n* = 166).

CRP respondents (*n* = 166) rated their perceptions of the importance of training across TS competencies. The five-point Likert Scale ranged from *very unimportant* to *very important*. Figure 1 shows the frequency of respondents who scored each TS competency as *important or very important.* Data revealed high scores across all competency categories.

**Figure 2.**
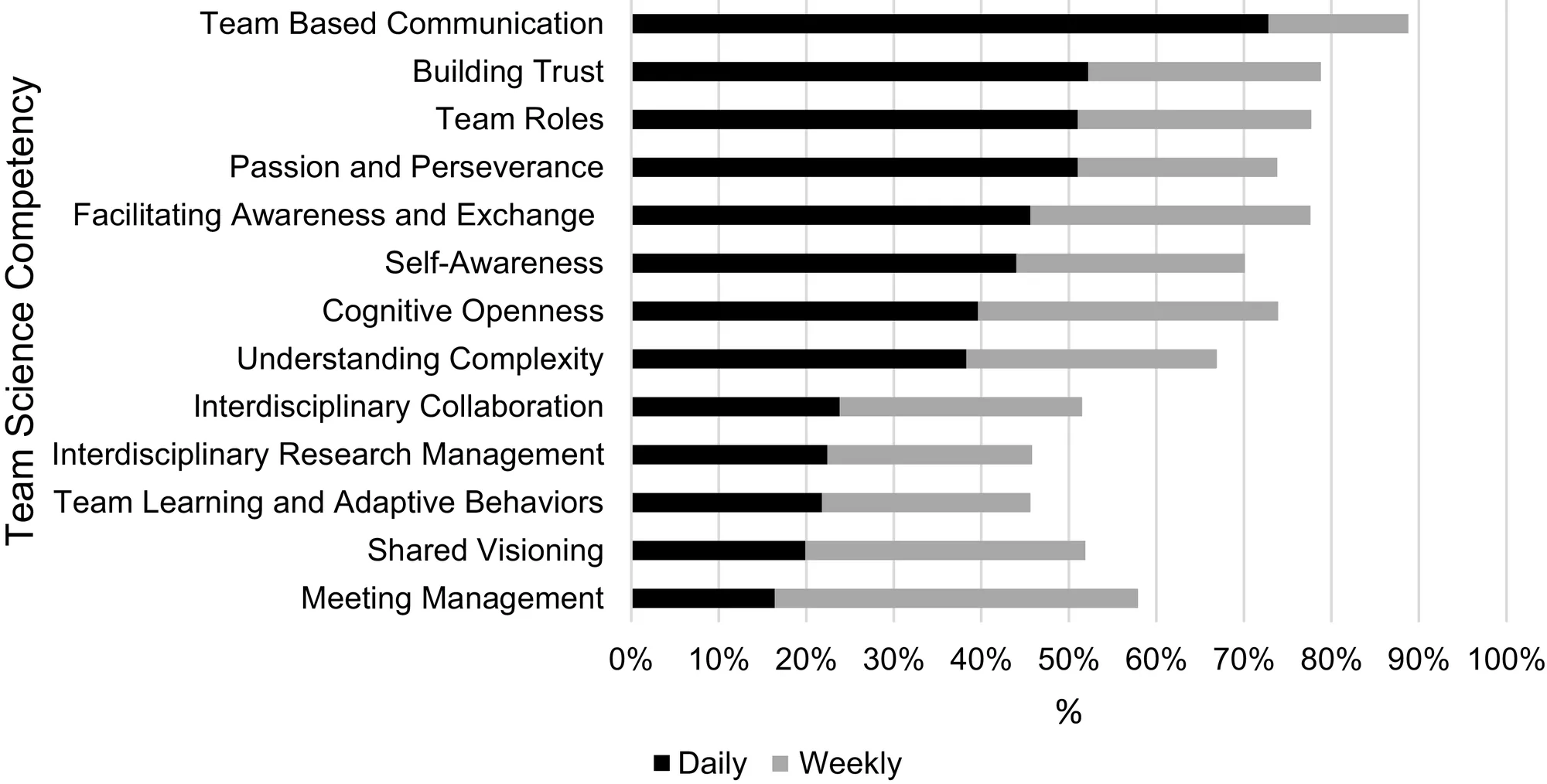
Percentage of CRP respondents that use TS competencies daily or weekly (*N* = 156).

We also sought to understand CRP’s perceptions of how often they used the TS competencies in their roles. Figure 2 illustrates that CRP respondents were using the TS competencies either daily or weekly.

**Figure 3.**
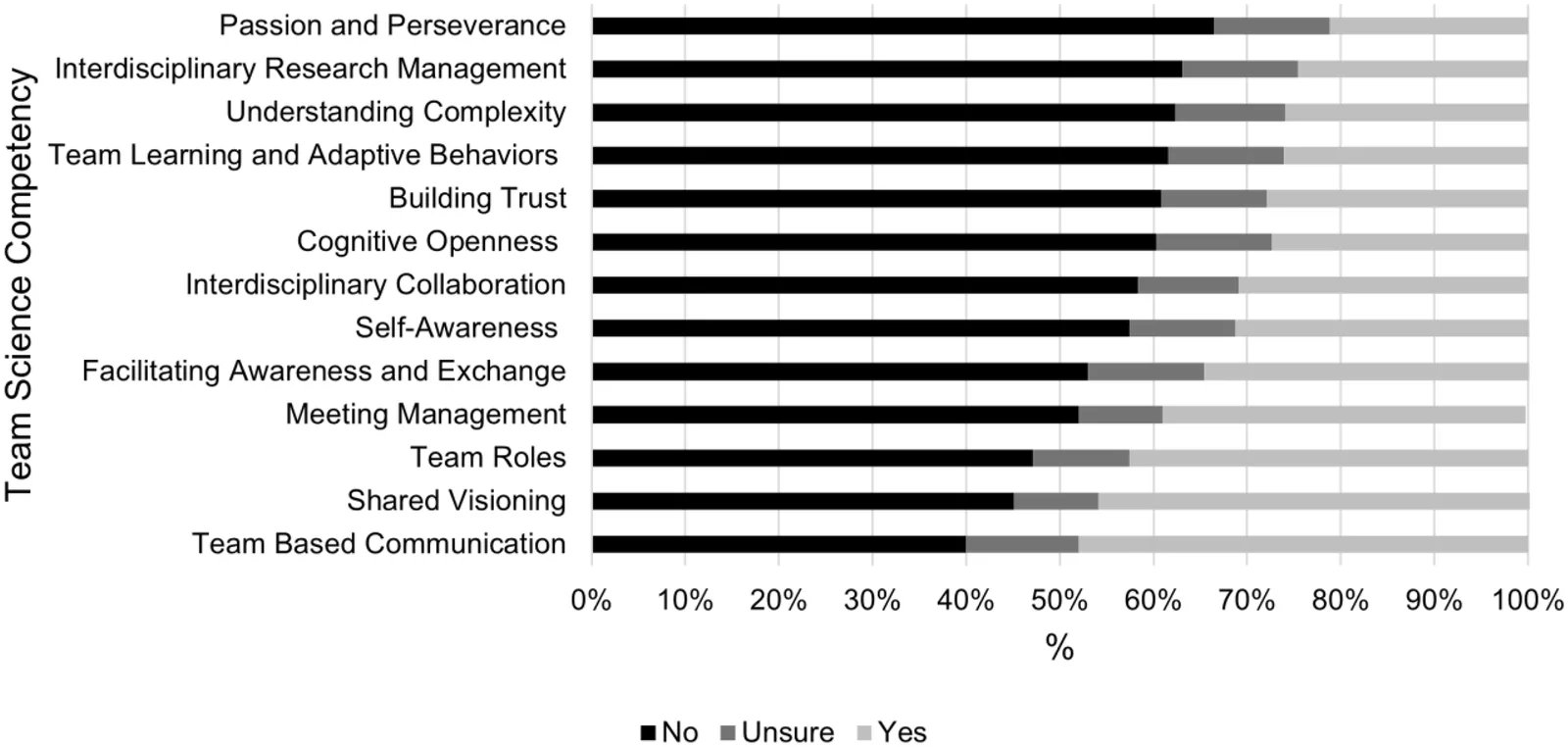
Figure 3 long description. Percentage of CRP respondents reporting prior training in team science competencies (*n* = 153).

Less than 46% of respondents report targeted training in the various TS competencies. Furthermore, over 10% were unsure if they had received training in specific TS competencies. Overall, the competencies “Team Roles” and “Shared Visioning” (42% and 46%, respectively) received the most training, followed by “Meeting Management (39%), (See Figure 3).

CRP respondents felt that future training in TS competencies would be beneficial to their roles; however, ‘Passion and Perseverance’ and “Interdisciplinary Research Management” and “Understanding complexity” were topics that had the least amount of prior training; where as “Team-Based Communication,” “Shared Visioning,” and “Team Roles” had the most prior TS training.

**Figure 4.**
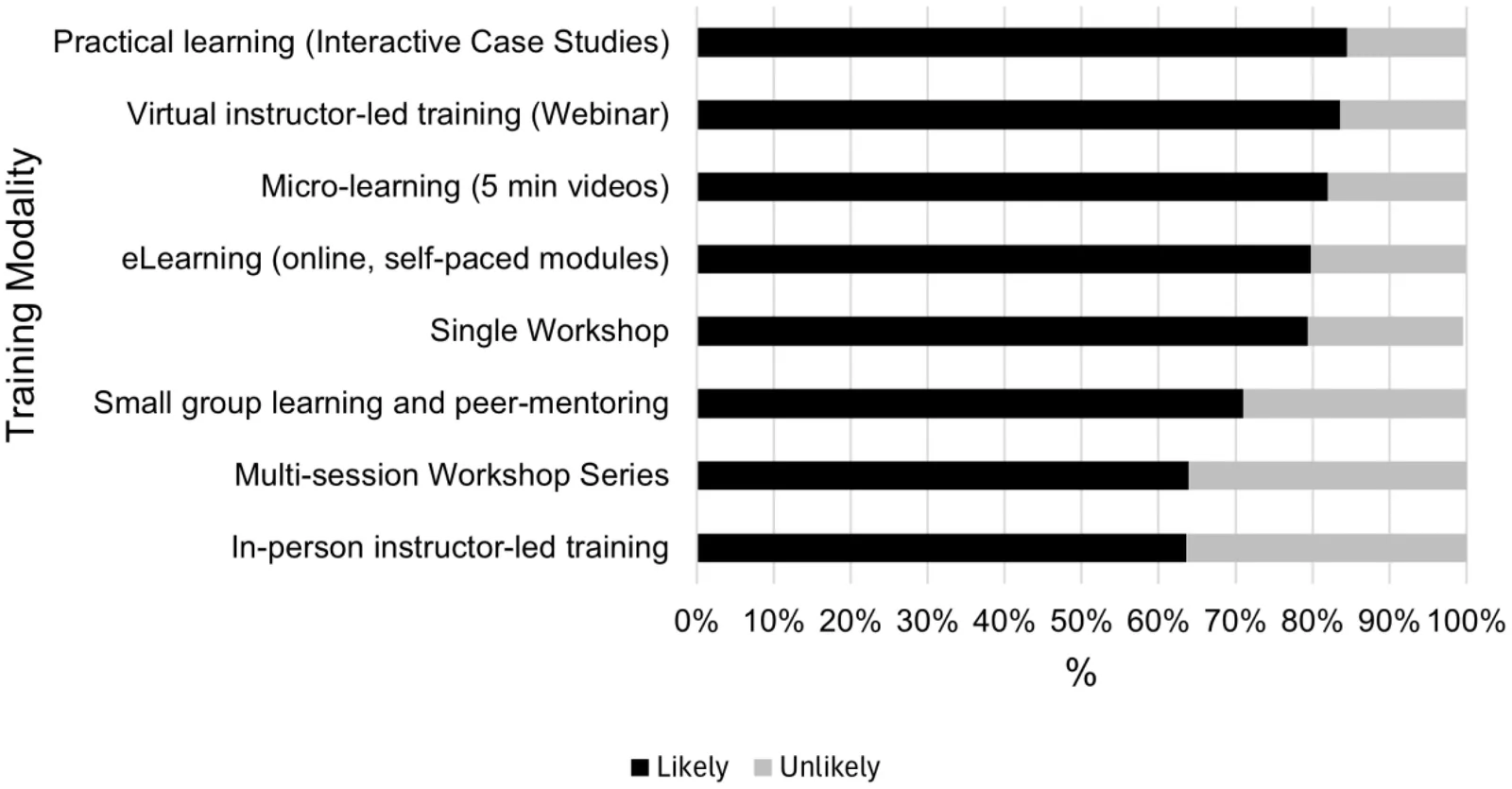
Figure 4 long description. Respondents’ preferences (%) for training modality (*n* = 143).

Respondents preferred less time intensive training and e-learning but also show preferences for interactive case-based training that have practical applications. Figure 4 illustrates CRP likeliness of attending specific training modalities.

### Focus group results

#### Demographics

A total of 72 survey participants volunteered their contact information for focus groups; however, only 22 individuals (31%) agreed to participate in 3 focus groups and 2 individual interviews. These contributed to the qualitative findings, representing a range of roles and experience levels within the clinical research workforce.

#### Focus group findings

What emerged was not a single, unified story, but something more nuanced. Participants spoke with clarity about what TS should be and with equal honesty about where it begins to fray in practice.

### Shared vision

Participants consistently described TS as a collective effort grounded in shared goals. At its best, it was described as “working collaboratively…each person filling their own unique roles… to make the job easier.” There was a strong sense that effective teams depend on recognizing the distinct contributions of each role, coordinators, nurses, investigators, and regulatory staff, each operating within their scope, but aligned toward a common purpose.

Yet, that alignment was not always easy to locate. Several participants described the “team” as expansive, sometimes overwhelming. One participant reflected: “It’s everybody, from clinicians to marketing (recruiting) to the medical assistant who checks on patients. . . I can tell you who makes up my immediate team, but my broader team… it’s huge.” For newer CRPs, this breadth translated into uncertainty. TS, while conceptually intuitive, was not always operationally defined. Knowing who is on the team, and more importantly who to turn to, was often learned informally over time.

### Communication

If shared vision was the ideal, communication was the mechanism that either sustained it or quietly unraveled it. Participants repeatedly returned to the idea that communication is foundational. Strong teams were described as those where “everyone knows they have a seat at the table. . . like your opinion matters.” Open dialog, mutual respect, and active listening were seen not as abstract ideals, but as daily necessities.

When communication faltered, the consequences were immediate. One participant described the ripple effect succinctly: “Any time we run into conflict . . . the timeline is longer. We thought we’d get something done by Friday. Now it’s next Friday.”

Another highlighted the subtle delicateness of communication dynamics. “She viewed my questioning as not trusting her.” In that moment a simple request for clarification became something else entirely, revealing how easily communication can shift from collaboration to defensiveness when norms are unclear or trust is uneven.

### Collaboration

Collaboration was universally valued but rarely described as simple. Participants emphasized that effective collaboration requires structure: timelines, coordination, and clear expectations across roles. Without that foundation, even well-intended collaboration can stall. As one participant stated: “Collaboration can right your ship or sink your ship…. If you’re waiting six weeks for a response, everything gets delayed.”

There was also recognition that collaboration extends far beyond immediate teams. CRPs described working across departments, disciplines, and systems, often simultaneously, which required not just coordination but a kind of cognitive flexibility: the ability to navigate multiple workflows, priorities, and communication styles at once. More experienced participants spoke about collaboration in systems terms, processes, timelines, and institutional coordination while less experienced participants framed it more interpersonally, focusing on access, responsiveness, and clarity.

### Role clarity

Role clarity surfaced as a persistent, and often invisible challenge. Participants described environments where responsibilities were loosely defined or constantly changing. In those spaces, understanding roles became something learned through experience rather than explicitly communicated.

As a participant noted: “It’s knowing who to go to. . . who would have the best answer . . . who’s doing what roles.” For early career CRPs, this caused navigating uncertainty through trial and error. For more experienced CRPs, it underscored the need for stronger systems, clearer workflows, defined responsibilities, and intentional onboarding processes. Without that clarity, inefficiency is maximized and at times introduces risk.

### Training and professional development

Across all groups, participants expressed a clear and consistent desire for training, especially in TS competencies. Yet, training was rarely experienced as a structured or supported activity. Instead, it was something individuals carved out for themselves. As one participant reflected: “I have to squeeze some things in sometimes after hours . . . you still have to get your work done.” Another was even more direct: “I’ve just made the time to do it. . . But I don’t think that’s the way it should be.”

There was also a clear mismatch between training formats and real-world constraints. Longer virtual sessions were often described as ineffective: “It’s a four-hour (virtual) session, . . .and really, I’ve been working the whole time through it.” In contrast, shorter, more focused formats, such as microlearning and case-based training were seen as more feasible and engaging. At the same time, participants expressed a longing for in-person interaction: “After four years of virtual thing . . . I’d love some in-person training. . . I’m craving that collaboration.” Yet even that desire was tempered by the reality: time constraints, staffing shortages, and competing demands.

### Institutional support

Underlying many of these experiences was a broader structural issue: inconsistent institutional support. Participants described limited protected time for training, lack of centralized communication about opportunities, and minimal integration of professional development into role expectations. One participant captured this gap succinctly: “We don’t get reminders about continuing education . . . that would be really cool from an institutional level.” In response, many CRP’s assumed personal responsibility for their own development, demonstrating initiative, but also highlighting a system that relies heavily on individual effort rather than a valued organizational design.

### Reframe team science

Perhaps one of the most striking insights came when a participant pushed back against the language often used to describe TS: “Team science sometimes gets lumped in with soft skills . . . and I feel very strongly that it is not.” This sentiment echoed across discussions. TS was not viewed as peripheral or optional, but as central, complex, and essential. It is the infrastructure that enables clinical research to function, often invisible, often under supported, but fundamentally necessary.

## Discussion

Our study focused on understanding the TS learning needs of CRPs and determining their self-perceived level of confidence in applying TS principles. These insights will help guide the development of future tailored TS training programs aimed at enhancing the success and efficiency of clinical research projects. This is the first needs assessment of CRP TS learning needs conducted to best plan future TS training. These data revealed a significant gap in formal training related to critical areas of TS with most CRPs experiencing limited to no training across several key TS competencies. By addressing the training disparities highlighted in our study and using flexible educational approaches, future trainers can enhance the ability of CRPs to fulfill their roles in team-based research.

Our survey data indicate that CRPs in the post-COVID-19 era prefer to receive TS training remotely compared to their previous expressed preferences for how to receive clinical research training (in-person) [26]. Moreover, they prefer a variety of training modalities, including case-based approaches, and though remote learning, including micro-learning as a delivery modality. Yet, they also desired more in-person training in addition to distance-based training, despite time and role constraints.

The analysis of the focus group data highlights communication as the central driver of effective teamwork, with participants emphasizing open dialog, active listening, and the use of multiple communication modes to support shared understanding. Successful collaboration was consistently linked to team-wide buy-in, an intentional shared vision, and recognition that all members contribute meaningfully regardless of hierarchy. Clear role definition and structured responsibilities further support effective functioning, particularly within small or interdisciplinary teams.

Professional development emerged as a valued priority, with training integrated into meetings and protected learning time. At the same time, respondents identified key challenges, including conflict management, navigating change, disengagement among remote team members, and the disproportionate impact of unresolved HR or individual behavioral issues on team dynamics. Overall, the findings portray TS as a people-centered process that flourishes through strong communication, inclusive leadership, and continuous learning, while remaining sensitive to interpersonal and organizational barriers.

Findings from both the quantitative and qualitative components converge to highlight a consistent gap. While CRPs recognize the importance of TS competencies and frequently apply them in practice, formal training remains limited. The integration of findings strengthens the interpretation that this is not simply a knowledge gap, but a structural gap in workforce development.

This study has several limitations. The purposeful sampling strategy limits the ability to calculate response rates and may introduce sampling bias. Institutional representation could not be determined due to the use of anonymity. Additionally, some survey items had missing responses because of optional question settings in the software. Future surveys should force responses to reduce skipped questions. By appending all 13 TS competencies to each survey question, the survey was long, likely contributing to survey fatigue. Although qualitative findings reached thematic saturation, smaller focus group sizes may limit the breadth of perspectives. Finally, while AI-assisted tools are supportive, their use remains an evolving methodological area. The use of Zoom provided an effective method for the study focus groups, allowing for participation from multiple institutions without reliance on travel.

### Recommendations to improve CRP team science training include:

- Workshops and Webinars: Given the notable interest, multisession workshops and webinars could be included in annual training plans to complement ongoing professional development efforts. These sessions are vital for reinforcing TS competencies. Discussions in focus groups indicated that these mechanisms may also be fraught with competitions for time (e.g., multi-tasking on other things during the learning process).
- Opportunities for Distance Learning and Microlearning Modalities: In response to the evolving educational landscape, offering hybrid or distance-learning opportunities, including bite-sized learning opportunities (e.g., online microlearning), could significantly enhance CRP engagement and retention of TS principles in keeping with busy adult learners.
- Encouragement of Institutional Support: We advocate for institutional support for CRPs to pursue educational opportunities for their roles, the science, and interdisciplinary TS. By directly addressing the training disparities highlighted through our focused study on the learning needs and TS competence of CRPs, institutional support of training can significantly bolster their ability to contribute to high-performing, interdisciplinary research teams. Actively promoting and supporting the professional development of CRPs not only benefits individual careers but also strengthens the overall success of TS efforts and study results. CRPs should be encouraged to attend TS trainings with the larger clinical research enterprise (e.g., with diverse investigators and trainees) to enhance self-identification of larger team membership.
- Assessment of Training Impact: Conducting follow-up studies to evaluate the impact of training on research outcomes, team dynamics, and individual career progression supports an evidence-based approach to professional development.

## Conclusion

As we continue to advance our understanding of the complexities and advantages of interdisciplinary team-centered clinical research, we must invest in equipping all members of these teams, especially CRPs, with the necessary skills and competencies to thrive and contribute to meaningful, impactful research. Through ongoing engagement and support, we can ensure that CRPs are valued and recognized as vital contributing members of the translational science community. This study will contribute to planning future research on this topic and TS training for CRPs.

## Figure 1. Long description

Horizontal bar chart displaying percentages of clinical research professionals (n = 166) who rated 13 team science competencies as either important (gray bars) or very important (black bars). Team-based communication had the highest endorsement, with nearly all respondents rating it important and more than 80% rating it very important. Team learning and adaptive behaviors, building trust, and self-awareness also received high ratings. Facilitating awareness and exchange, cognitive openness and intersubjectivity, understanding complexity, team roles, interdisciplinary collaboration, and interdisciplinary research management were rated important by approximately 85%–95% of respondents, with very important ratings generally between 50% and 70%. Meeting management, shared visioning, and passion and perseverance received the lowest very important ratings (approximately 40%–47%), although about 85%–90% still rated these competencies as important. Overall, respondents viewed all team science competencies as important, with communication and collaborative behaviors receiving the strongest endorsements.

Navigate back to Figure 1..

## Figure 3. Long description

Figure 3 presents a horizontal stacked bar chart of prior training in team science competencies among 153 Clinical Research Professionals. Competencies include Passion and Perseverance, Interdisciplinary Research Management, Understanding Complexity, Team Learning and Adaptive Behaviors, Building Trust, Cognitive Openness, Interdisciplinary Collaboration, Self-Awareness, Facilitating Awareness and Exchange, Meeting Management, Team Roles, Shared Visioning, and Team-Based Communication. Each bar is divided into No (black), Unsure (gray), and Yes (light gray). Across all competencies, the majority of respondents reported no prior training, with the highest percentages (approximately 60%) for Passion and Perseverance, Interdisciplinary Research Management, Understanding Complexity, Team Learning and Adaptive Behaviors, and Building Trust. Reports of prior training were highest for Team-Based Communication, Shared Visioning, and Team Roles (approximately 35–45%) and lowest for Passion and Perseverance and Interdisciplinary Research Management (approximately 20%). Unsure responses remain relatively stable across competencies at roughly 10–20%. The overall pattern indicates limited prior training in team science competencies among respondents.

Navigate back to Figure 3..

## Figure 4. Long description

Figure 4 presents respondents’ preferences for training modalities (n = 143) using horizontal stacked bars. Each bar displays the percentage reporting a modality as Likely (black) or Unlikely (gray). Practical learning through interactive case studies is the most preferred modality, with approximately 85% likely and 15% unlikely. Virtual instructor-led training (webinars) is similarly preferred, with about 85% likely participation. Micro-learning formats consisting of 5-minute videos are preferred by roughly 80–85% of respondents, while eLearning through online self-paced modules and single workshops are each preferred by about 80%. Small-group learning and peer mentoring is viewed favorably by approximately 70% of respondents. Multi-session workshop series and in-person instructor-led training receive the lowest levels of interest, with about 60–65% reporting they are likely to participate and 35–40% reporting they are unlikely. Overall, respondents favor practical, flexible, and online learning approaches over more time-intensive or in-person training formats.

Navigate back to Figure 4..

## Table 1. Long description

Table 1 presents 13 Team Science competencies identified for Clinical Research Professionals (CRPs). The competencies include building trust, meeting management, interdisciplinary collaboration, understanding complexity, team learning and adaptive behaviors, team-based communication, shared visioning, passion and perseverance, team roles, self-awareness, interdisciplinary research management, cognitive openness, and facilitating awareness and exchange. Abbreviation: CRP = Clinical Research Professional.

Navigate back to Table 1..

## Table 2. Long description

An image layout combining text and illustrative design elements. At the top, a prominent stylized heading serves as the central focal point, drawn with bold lines and geometric accents. Directly beneath the heading, decorative divider lines frame a centered block of body text arranged in a clean, multi-line paragraph structure. To the sides of the text, minimal vector graphic elements—such as subtle abstract shapes and geometric icons—provide visual balance to the frame. The overall composition uses high contrast between the background and text elements to ensure high legibility and a clear visual hierarchy from the top title down to the descriptive text.

Navigate back to Table 2..

## Table 3. Long description

This visual consists of a single bordered box containing nine numbered qualitative survey or interview questions, split across two distinct sections by a horizontal line. The upper portion, titled “A. Questions Related to Survey Results,” contains three questions set in italics. The first question asks what team science means to the respondent, including who makes up the research team. The second question references survey findings showing that respondents needed more information on interdisciplinary research management and collaboration, asking what those concepts mean to the participant. The third question notes that shared visioning caused confusion in the group and asks for the participant's understanding of the term. The lower portion, titled “B. Questions Related to You and Your Team,” contains six additional italicized questions focused on workplace dynamics and professional development. The first question asks for a description of a recent situation where the team worked well together and the factors contributing to that success. The second asks how the team handles disagreements or conflicts and requests a specific instance. The third asks the participant to recall a time when the team did not work well together, identifying the main challenges encountered. The fourth asks how those challenges affected team performance and project outcomes. The fifth asks how the participant balances workload with ongoing training and professional development needs. Finally, the sixth question notes a survey trend toward online training, live webinars, and asynchronous on-demand approaches, asking for the participant's thoughts on those specific modalities.

Navigate back to Table 3..
